# Incidental Finding of Microfilaria in Lymph Node Cytology: A Case Report

**DOI:** 10.7759/cureus.31275

**Published:** 2022-11-08

**Authors:** Bhavinee Pathak, Sabiha Maimoon

**Affiliations:** 1 Pathology, NKP (Narendra Kumar Prasadrao) Salve Institute of Medical Sciences, Nagpur, IND

**Keywords:** incidental, case report, cytology, lymph node, microfilaria

## Abstract

Filariasis is a global public health problem. We routinely examine it in peripheral smears made from samples collected during the night. Though the prevalence is high, microfilariae are rarely found in cytology smears. A lymph node presenting as a filarial nodule is an unusual occurrence. Also, lymph node fine needle aspiration cytology (FNAC) with a finding of microfilariae is uncommon. We would like to report the case of incidental microfilariae finding in a patient who presented with a supraclavicular lymph node mass. The patient presented with a right supraclavicular lymph node mass of size 2 cm × 2 cm and severe fever during the night for six months. The USG-neck region revealed enlarged lymph nodes in the right supraclavicular region. She was provisionally diagnosed as a case of cervical lymph node swelling under evaluation. However, on ultrasound-guided FNAC of the lymph node, microfilariae were found incidentally. In cases where clinical presentations of filariasis are absent, FNAC may aid in the diagnosis of microfilariae in the lymph node.

## Introduction

Filariasis is a major global public health problem and is endemic in India [1]. The causative agents are the nematode worms *Wuchereria bancrofti* and *Brugia malayi*. It is the Culex mosquito that transmits the disease [2]. Routinely, the diagnosis is made by the demonstration of microfilaria in three consecutive night peripheral smears [3]. Despite the high incidence in India, the occurrence of filarial nodules and their diagnosis by lymph node fine needle aspiration cytology (FNAC) is unusual [4]. In our case, the FNAC of the right supraclavicular lymph node revealed an incidental finding of microfilaria without any clinical features of filariasis or microfilaremia.

## Case presentation

Patient information

A 37-year-old female presented with chief complaints of swelling in the right side of the neck and fever during the night for the last six months. It was gradual in onset. The swelling progressively increased in size. She also had complaints of pain in the right side of her neck and generalized weakness. Her menstrual cycle was regular with an average flow.

Clinical findings

Local examination revealed a palpable lump of size 2 cm × 2 cm present over the right supraclavicular region. It was firm, non-tender, and mobile. It was globular in shape. There were no skin changes over the lump. There was no bleeding, discharge, or local rise in temperature. The systemic examination was within normal limits.

Timeline of the current episode

The patient had been complaining of swelling on the right side of the neck and an evening rise in temperature for the last six months. She described feeling sharp tingling sensations in the swelling during the night. The patient had generalized weakness. She gave a history of weight loss and loss of appetite in the last two to three months. There was no history of trauma, diabetes mellitus, bronchial asthma, tuberculosis, or hypertension.

Diagnostic assessment

The USG neck revealed a few (2-3) subcentimetric and enlarged lymph nodes in the right supraclavicular region, the largest of which measured 1.7 mm × 9 mm. Few of them showed loss of fatty hilum. The complete blood count was normal. The peripheral smear examination revealed a normal count and morphology of cells with no abnormal findings. ESR was 19 mm/hr. The patient was given a provisional diagnosis of a case of cervical lymphadenopathy under evaluation.

The patient was then advised to undergo USG-guided FNAC. The aspirate was scanty and hemorrhagic. The smears were examined under a microscope.

Diagnosis

**Figure 1:**
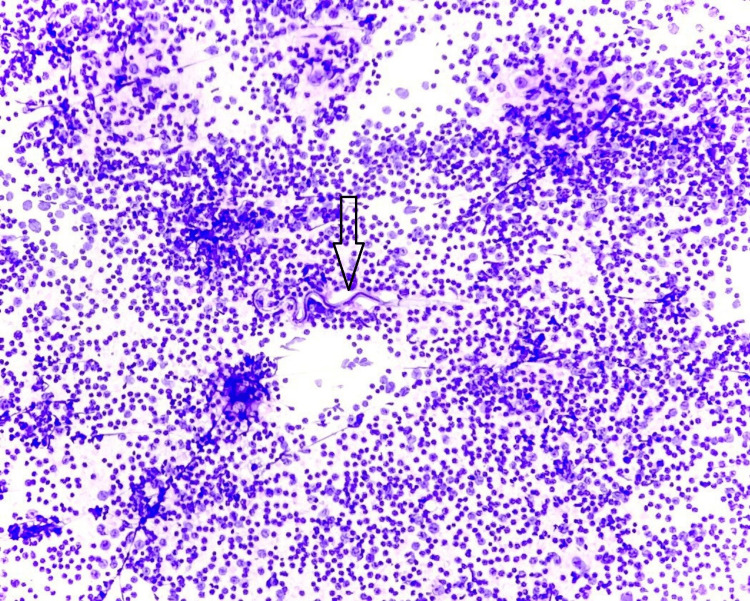
Haematoxylin and eosin stain; 10× power showing cellular smear with mixed cell population.

**Figure 2:**
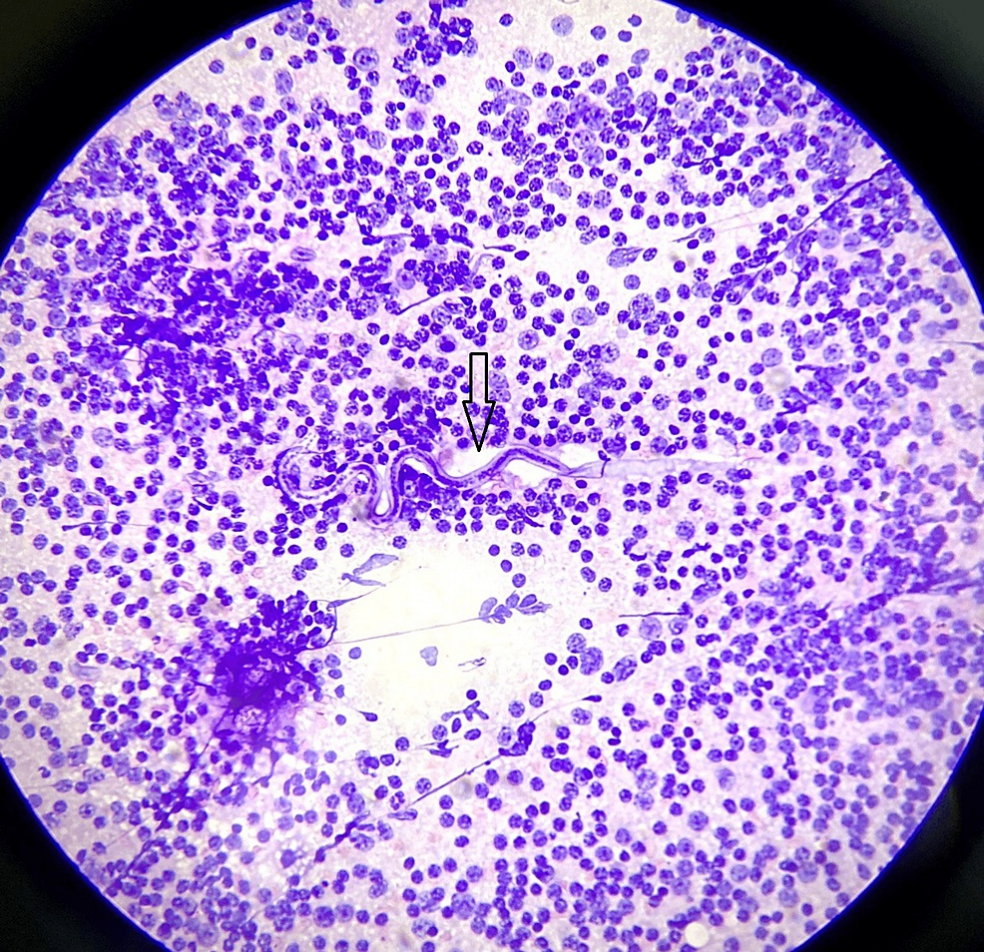
Haematoxylin and eosin stain; 40× power showing coiled and slightly curved microfilaria.

A diagnosis of filariasis with reactive lymphadenopathy was given on the basis of lymph node mass cytology. In the patient, FNAC lymph node smears were cellular (as can be seen in Figure 1). They revealed a mixed cell population of small and large lymphoid cells, with predominantly mature lymphocytes, many histiocytes, tingible body macrophages, and occasional eosinophils. An ensheathed, coiled, and slightly curved microfilaria was observed (Figure 2). The background contained red blood cells (RBCs).

## Discussion

The number of people estimated to harbour microfilaria is millions, and a significantly high number of people suffer from disease manifestations of filaria [5]. Filariasis cases are widely distributed in the tropics and subtropics of sub-Saharan Africa, Southeast Asia, India, and the Pacific Islands. The highest number of cases were reported in India. Filariasis is a term for infection caused by any of the filarial worms; however, in daily practice, the term filariasis is indicative of lymphatic filariasis caused by Wuchereria or Brugia species. The vector used for transmission was the Culex mosquito. Microfilaria exhibit nocturnal periodicity in the peripheral blood. It presents as elephantiasis in the late stages of the disease. Peripheral blood examination shows motile microfilariae parasites in nighttime blood samples collected between 10 p.m. and 4 a.m. [6].

The circulating filarial antigen (CFA) detection test is now regarded as the gold standard for the demonstration of microfilarial organisms. There are limited reports in the literature addressing the importance of FNAC as a tool for diagnosing filariasis [7]. FNAC is not routinely performed for the diagnosis of clinically suspected filariasis. However, incidental detection of fine-needle aspiration cytology smears has been reported [8,9]. In cases of elephantiasis, occult filariasis, lymphangitis, and early stages of allergic presentations, microfilariae may not be seen on peripheral blood film examination [2,10].

There are rare case reports of incidental findings of microfilariae in cytology smears of thyroid nodules, bronchial aspirates, axillary lymph nodes, pericardial fluid, and breast lesions [3]. Our patient had right-sided supraclavicular lymph node swelling without any clinical presentation of filariasis.

In our case, the presence of microfilaria in the lymph node FNAC was an incidental finding, highlighting the importance of thorough screening in patients in whom clinical manifestations of filariasis and microfilaremia in the blood are absent [11].

## Conclusions

In endemic areas such as India, the association of microfilariasis should be considered. Fine needle aspiration cytology can be a sensitive, cost-effective, and invaluable tool for the detection of helminthic aetiology in unexplained lymph node masses. This emphasises the importance of careful screening of cytology smears to detect microfilaria in patients with no clinical evidence or microfilaremia. This will help provide accurate and timely treatment to patients.
